# A Comparative Study of Conventional Physiotherapy versus Robot-Assisted Gait Training Associated to Physiotherapy in Individuals with Ataxia after Stroke

**DOI:** 10.1155/2018/2892065

**Published:** 2018-02-20

**Authors:** Marcia Belas dos Santos, Clarissa Barros de Oliveira, Arly dos Santos, Cristhiane Garabello Pires, Viviana Dylewski, Ricardo Mario Arida

**Affiliations:** ^1^Physiology Department, Universidade Federal de São Paulo (UNIFESP), São Paulo, SP, Brazil; ^2^Physiotherapy Department, Associação de Assistência a Criança Deficiente (AACD), São Paulo, SP, Brazil; ^3^Faculty of Medicine, University of Sao Paulo (USP), São Paulo, SP, Brazil

## Abstract

**Objectives:**

To assess the influence of RAGT on balance, coordination, and functional independence in activities of daily living of chronic stroke survivors with ataxia at least one year of injury.

**Methods:**

It was a randomized controlled trial. The patients were allocated to either therapist-assisted gait training (TAGT) or robotic-assisted gait training (RAGT). Both groups received 3 weekly sessions of physiotherapy with an estimated duration of 60 minutes each and prescribed home exercises. The following outcome measures were evaluated prior to and after the completion of the 5-month protocol treatment: BBS, TUG test, FIM, and SARA. For intragroup comparisons, the Wilcoxon test was used, and the Mann–Whitney test was used for between-group comparison.

**Results:**

Nineteen stroke survivors with ataxia sequel after one year of injury were recruited. Both groups showed statistically significant improvement (*P* < 0.05) in balance, functional independencein, and general ataxia symptoms. There were no statistically significant differences (*P* < 0.05) for between-group comparisons both at baseline and after completion of the protocol.

**Conclusions:**

Chronic stroke patients with ataxia had significant improvements in balance and independence in activities of daily living after RAGT along with conventional therapy and home exercises. This trial was registered with trial registration number 39862414.6.0000.5505.

## 1. Introduction

Stroke is the third most common cause of death and the biggest factor for disability in adults of developing nations, just behind cancer and heart diseases [1]. Approximately 795,000 stroke cases occur every year in the USA [2] with 2-3% in the cerebellum area [3]. The loss of motor skills is one of the most common complaints of stroke survivors as approximately 75% of these patients have some walking disability that could result in high risk of falls [4, 5]. In a prospective study, 256 stroke patients were evaluated in the acute phase, with approximately 27% reporting at least one fall in a three-month period [6].

Impairment in the posterior circulation that involves the cerebellum or brainstem region may lead to damages in several important functions, such as balance, movement coordination, speech, hearing, ocular movement, and swallowing [7, 8]. Ataxia is an important sequela observed and recognized for its presentation as a loss of coordination, dysmetria, dysarthria, hypotonia, rebound phenomenon, and nystagmus [9]. Gait ataxia is described by a stumbling walking pattern, an irregular foot placement, an increased step, an enlarged stance, and an abnormal joint torque [10, 11].

When the depletion of balance ability is associated with decreased joint mobility, muscle tone problems, and loss of proprioception, there is an increase in the difficulty to perform activities of daily living for individuals with stroke injury [12]. Consequently, balance training is crucial for rehabilitation treatment. Conventional gait therapy (CGT), such as the Bobath concept, proprioceptive neuromuscular facilitation, therapist-assisted walking, and the use of braces or other devices are common treatment approaches [13]. Furthermore, high severity stroke patients with poor coordination in walking may benefit from treatment with a robotic device that allows task-focused training [14].

Robot-assisted gait training (RAGT) has been used since 1980 to assist patients with dysfunction in movement caused by neurological disorders [15]. This treatment is based on the body weight-supported treadmill (BWSTT) principle and achieves functional motor relearning through the repetitive practice of all different phases of gait [16]. Training the same movement repetitively enables the nervous system to develop circuits for better communication between the motor center and sensory pathways [17].

Treatment by RAGT compared with conventional treatment on the treadmill presents advantages, including training duration, more reproducible symmetrical gait patterns, operation by a single therapist, and a reduction in the energy expenditure imposed upon the therapists [18]. In addition, recent research revealed that robot-assisted treadmill training resulted in a more symmetrical muscle activity pattern in paretic patients compared with conventional treatment [5]. It is important to highlight that there have been very few investigations on RAGT in individuals with ataxia poststroke.

A systematic review on the efficacy of rehabilitation robotics for walking training in neurological disorders showed that patients injured by stroke had statistically significant amelioration in walking speed, functional abilities, and motor functions after treatment [19]. However, a study that compared treadmill training with both partial body weight support and robotic training revealed that therapy with partial body weight support was superior to robotic training for subjects with chronic stroke [20].

One of the possible drawbacks of treatment with RAGT may be the excessive passive guidance of device, which could potentially reduce patient effort and treatment effectiveness [21]. Additionally, another important factor is the patients' limited degree of freedom with the equipment, which could result in abnormal torque [18].

Considering the critical importance of the subject as well as the shortage and inconclusive information about it, this study aimed to assess the influence of robot-assisted gait training on balance and coordination and to verify the functional independence in activities of daily living of chronic stroke survivors with ataxia after at least one year of injury.

## 2. Methods

### 2.1. Subjects

This was a randomized controlled trial approved by the Universidade Federal do Estado de São Paulo (UNIFESP) ethics committee (number 933.112). All the patients who participated or their legal representatives gave written informed consent voluntarily without financial gains.

The inclusion criteria were stroke survivors with ataxia, minimum time of injury over 1 year (in the chronic phase of rehabilitation), cerebellar stroke verified by an initial MRI, clinical stability, presence of hemiplegia or quadriplegia motor impairment, and admission to the Associação de Assistência a Criança Deficiente (AACD) Rehabilitation Center from September 23, 2014, to December 20, 2015. Patients from both genders who were at least 18 years of age were accepted. A physician examined all the patients and described their diagnosis in medical records.

The exclusion criteria were physical disability that made training with the robotic device unsafe such as cognitive impairment, dementia, aphasia, presence of other orthopedic or neurosurgical problems in the lower extremities, pressure ulcers on the hips or lower extremities, weight higher than 120 kg, ataxia originated by progressive disease, and not accomplishing the proposed treatment.

The participants were allocated to each arm of this study using a weekly timetable, which illustrated a list of sessions available for the proposed treatment. This specific schedule was formulated given ten different schedule options per week. Depending on their preferable time session chosen, they were allocated to one of the two arms of this study: therapist-assisted gait training (TAGT) or RAGT. Each participant completed three sessions per week. Furthermore, their timeline was organized with the availability and capacity of the institution for both proposed interventions.

### 2.2. Evaluation Protocol

All participants were examined by a blinded evaluator at two-time points: before and after the protocol treatment was completed. The Berg Balance Scale (BBS), the Functional Independence Measure (FIM), the Timed Up and Go Test (TUG), and the Scale for the Assessment and Rating of Ataxia (SARA) were the tools used for baseline and outcome evaluation.

The BBS test is used to evaluate balance and risk of fall. The BBS focuses on static and dynamic balance, which includes 14 tasks with a maximum score of 56 [22, 23].

The TUG test evaluates sitting balance, transfers from sitting to standing, stability in ambulation, and gait course changes [24].

The FIM is used to assess neuropsychological and motor disability. This functional scale includes 18 items allocated into 6 domains: 2 cognitive and 4 motors. Each item has an increasing score from 1 to 7 (1 = maximum of functional dependence and 7 = maximum of functional independence). The minimum value of the entire range is 18, and the total score is 126 [22].

The SARA is used to assess the severity and treatment effectiveness of cerebellar ataxia. Some studies have shown its usefulness in stroke individuals [25, 26]. The total score ranges from 0 (no ataxia) to 40 (severe ataxia), and it is composed of the following 8 items [27]: 1 gait (0–8); 2 stance (0–6); 3 sitting (0–4); 4 speech disturbance (0–6); 5 finger chase (0–4); 6 nose finger test (0–4); 7 fast alternating hand movement (0–4); and 8 heel-shin slide (0–4); the four extremities are evaluated bilaterally, and the mean values are used to calculate the total score [26].

### 2.3. Training Protocol

The patients were allocated into two groups according to the treatment received for gait training: therapist-assisted gait training (TAGT) and robot-assisted gait training (RAGT). Both groups were submitted to 3 intensive sessions of physiotherapy per week and were prescribed home exercises. Each group had 2 sessions of conventional physiotherapy and 1 session of gait training with an estimated duration of 60 minutes each. The TAGT was delivered over ground with the use of a walker device, if necessary. The RAGT was performed with a robot-driven exoskeleton orthosis equipment Lokomat® 5.0. In an automated electromechanical gait rehabilitation, the Lokomat device consists of a robotic gait orthosis combined with a harness-supported body weight system used in combination with a treadmill. The estimated protocol time was 5 months.

For both groups, the rehabilitation program consisted of muscle stretching and strengthening, balance training, postural stability control, sensory techniques, and functional daily activities. Furthermore, the patients were encouraged to continue practicing exercises at home. The professional in charge of the physiotherapy treatment was very knowledgeable and experienced in treating neurological disorders.

The main aim of RAGT was to improve the quality of movement and the coordination of both legs. The parameters were performed at a low speed (between 0.8 kph and 1.5 kph) and were adjusted gradually according to the patients' evolution. The body weight support was 50% of the patient weight at the beginning of the protocol treatment, which was gradually reduced until a minimum of 10% at the end the protocol training.

### 2.4. Statistical Analysis

All parametric results are illustrated as the mean ± standard deviation for each group in the tables and text. A level of significance of *P* < 0.05 was accepted for this study. The data were analyzed using change scores from pre- to posttraining with intragroup and between-group comparisons. Only data from subjects who completed the protocol training were used. The demographic characteristics (age and median onset time) were analyzed using the Mann–Whitney *U* test. Gender, the distribution of diagnosis, and side motor impairment were analyzed using the two-ratio test. The Wilcoxon test was used for intragroup comparisons, and the Mann–Whitney *U* test for between-group comparison. The data were analyzed using SPSS software V17, Minitab 16, and Excel Office 2010.

## 3. Results

A total of 19 patients were enrolled in this study from September 23, 2014, to December 20, 2015. The TAGT group contained 8 subjects. The RAGT group contained 11 subjects, of whom 4 were excluded for not complying with the protocol treatment criteria, leaving 7 subjects. The median age of all patients was 50.8 ± 13.3 years. The median onset time of all patients was 7.8 ± 4.8 years. The population studied was predominantly composed of hemorrhagic stroke patients (*P* = 0.017) (two-ratio test). The distribution of motor impairment side was homogeneous for both groups. The demographic distribution and baseline characteristics for each group are reported separately in Table 1.

After protocol treatment, both groups showed statistically significant improvement (*P* < 0.05) in balance, functional independence in daily living activities, and general ataxia sequela symptoms. Such betterment was evidenced by the intragroup comparison of all functional scales scores from pre- and posttreatment, which are described in Table 2.

**Figure 1:**
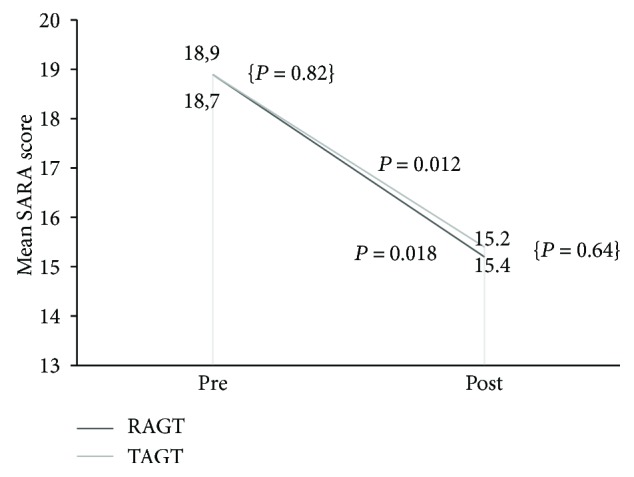
Changes in assessment and rating of ataxia. *P* value represents intragroup differences. Requirement for a statistically significant difference: *P* < 0.05. *P* value for between-group comparison: {*P* = 0.82; *P* = 0.64}. Requirement for a statistically significant difference: *P* < 0.05.

Figure 1 shows the difference in the means of the SARA score for both TAGT and RAGT at baseline and after the conclusion of the protocol treatment. There was a significant improvement in the SARA score at the completion of the protocol training for both groups.

Although the two groups had different mean values for the functional scale scores (Table 2), there was no statistically significant difference for the between-group comparison at both baseline and after completion of the protocol treatment (BBS pre *P* = 0.816, post *P* = 0.862; TUG pre *P* = 0.807, post *P* = 0.684; FIM pre *P* = 0.318, post *P* = 0.343; and SARA pre *P* = 0.817, post: *P* = 0.643).

## 4. Discussion

In general, the major goal of a rehabilitation program is to improve gait capacity, which has been directly connected to the quality of life in poststroke patients [28]. Moreover, other frequently addressed complaints are postural instability and balance impairments, which are related to a loss in independence to perform activities of daily living [29].

Balance involves a range of motor skills that are directly connected to sensory-motor processes, functional contexts, and the environment [30]. Stroke is frequently associated with the impairment of these abilities. However, there are few studies that have reported the effect of RAGT on balance [18]. This research aimed to assess the influence of robot-assisted gait training on balance and to verify the functional independence in daily living activities of chronic stroke survivors with ataxia after at least one year of injury.

In the present study, we used the BBS and TUG tests to assess balance control. The results indicated a significant improvement in balance for both RAGT and TAGT groups. These findings agree with previous reports [18, 31, 32]. Furthermore, when the balance outcomes were compared between groups, that is, RAGT versus TAGT, no significant difference was demonstrated, which is also supported by several studies [18, 32–35]. In contrast, Dundar et al. showed greater improvement in balance for the group that received RAGT; their treatment protocol included patients with a stroke onset ranging from 28 days to 365 days, which differs from the current study that included only subjects with chronic stroke [22].

After the protocol treatment was completed, both RAGT and TAGT groups revealed a significant improvement in mean FIM scale results, which indicates that both strategies could be effective in improving independence in activities of daily living. Moreover, no significant difference was encountered when these outcomes were compared between groups. Tong et al. also compared the therapeutic effects of TAGT and RAGT interventions [32]. Our findings agree with regard to the improvement in general independence and the absence of significant differences for the intergroup comparison. On the contrary, other studies presented statistically superior improvement in general activities for the group that received RAGT [22, 28]. This discrepancy in the findings is still relatively unexplained and should be investigated further.

In the present study, an improvement in the SARA outcome measure after protocol treatment was observed in stroke patients with ataxia sequela, showing a better control in postural disorders. When the total SARA scores were compared between groups, there was no statistically significant difference. Only a few studies have focused on the assessment and rehabilitation treatment of ataxia disorders and have demonstrated the beneficial effects of rehabilitation programs [36, 37]. Although there are published investigations of different techniques, such as virtual reality, biofeedback, and treadmill exercise with or without body weight support [36–39], none have evaluated the effect of RAGT on chronic stroke patients with ataxia.

Gait ability is closely associated with balance control [40]. A systematic review concluded that RAGT may increase the chance of recovering independent walking ability in poststroke patients [22]. The greatest improvement in independent walking and walking speed was observed during the early months after stroke [22, 41].

A multicenter randomized clinical trial that compared RAGT with TAGT interventions in subjects affected by subacute stroke showed that TAGT rendered better outcomes for gait improvement [33]. Similarly, another comparative study showed greater improvements in overground walking speed in chronic stroke survivors who received TAGT [20].

In the last two decades, several studies have demonstrated different results regarding the effects of RAGT treatment for gait rehabilitation on poststroke individuals [14, 31, 33, 42, 43]. Robotic assistance is a valuable resource for recovering gait ability. Notwithstanding, only a few studies have investigated its effectiveness for balance recovery in chronic stroke survivors. Additionally, there is no published study that has specifically focused on treating ataxia with RAGT. This study has addressed this gap in knowledge and has contributed to the evidence that robotic therapy may also be an additional asset for the balance treatment of ataxic patients.

This study has some limitations. In particular, some limitations are related to the small sample size. Moreover, future studies should evaluate a longer follow-up time and patients in acute and subacute stroke phases. Additionally, we recommend that future studies should address the analysis of RAGT in larger samples with a comparative measurement and include patients in all stages of stroke. Furthermore, we also suggest the investigation of supplementary interventional methods to gain a comprehensive understanding of the balance control mechanism in ataxic patients.

## 5. Conclusions

This study concluded that chronic stroke patients with ataxia sequela had a significant improvement in balance and independence in activities of daily living after treatment with RAGT along with conventional therapy and home exercises. For this sample size, the outcomes were similar regardless of the applied treatment strategy, that is, RAGT or TAGT. Both treatment approaches should be included as options in balance rehabilitation programs for ataxic patients.

## Figures and Tables

**Table 1:** Summary of demographic and baseline characteristics by intervention therapy.

Demographic and baseline characteristics	Therapist-assisted (*n* = 8) X (SD)	Robot-assisted (*n* = 7) X (SD)	*P* value
Mean age (year)	56.4 (11.8)	44.4 (12.7)	0.064
Gender, number (M/F)	6/2	5/2	0.876
Diagnosis, number (I/H)	2/6	2/5	0.876
Right side motor impairment, number	2	2	0.876
Left side motor impairment, number	2	2	0.876
Both side motor impairment, number	4	3	0.782
Onset (year)	10.5 (5.4)	4.8 (0.92)	0.021^∗^

*P* value represents between-group differences. ^∗^Requirement for a statistically significant difference: *P* < 0.05. SD: standard deviation; M: male; F: female; I: ischemic; H: hemorrhagic.

**Table 2:** Outcome measures at baseline and after protocol treatment by intervention therapy.

Functional scale	Therapist-assisted		*P* value	Robot-assisted		*P* value
	Pre	Post		Pre	Post	
BBS	27.3 ± 10.8	35.5 ± 14.1	0.012^∗^	26.6 ± 18.0	32.4 ± 18.8	0.018^∗^
TUG	0 : 28 ± 0 : 11	0 : 22 ± 0 : 10	0.017^∗^	0 : 46 ± 0.40	0 : 27 ± 0 : 17	0.011^∗^
FIM	80.9 ± 9.6	85.4 ± 8.2	0.016^∗^	73.9 ± 14.6	78.5 ± 12.9	0.042^∗^
SARA	18.9 ± 6.8	15.4 ± 5.6	0.012^∗^	18.7 ± 7.6	15.2 ± 6.8	0.018^∗^

All values are shown as the mean ± SD. *P* value represents intragroup differences. ^∗^Requirement for a statistically significant difference: *P* < 0.05. The nonparametric Wilcoxon test was used to compare pre- and posttreatment measurements. BBS: Berg Balance Scale; TUG: Timed Up and Go Test reported in seconds; FIM: Functional Independence Measure; SARA: Scale Assessment and Rating of Ataxia.
